# A nationwide cohort study of the association of benzodiazepines with SARS-CoV-2 infection and clinical outcomes

**DOI:** 10.1038/s41598-022-20335-z

**Published:** 2022-09-24

**Authors:** Hye Yoon Park, Junhyun Kwon, Suk Kyoon An, Eun-Cheol Park

**Affiliations:** 1 Department of Psychiatry, Yonsei Wonju University College of Medicine, Wonju, Republic of Korea; 2 Section of Self, Affect and Neuroscience, Institute of Behavioral Science in Medicine, Yonsei University College of Medicine, Seoul, Republic of Korea; 3 Institute of Health Services Research, Yonsei University, Seoul, Republic of Korea; 4 Department of Psychiatry, Yonsei University College of Medicine, Severance Hospital, 50-1 Yonsei-ro, Seodaemun-gu, Seoul, Republic of Korea; 5 Graduate Program in Cognitive Science, Yonsei University, Seoul, Republic of Korea; 6 Department of Preventive Medicine, Yonsei University College of Medicine, 50-1 Yonsei-ro, Seodaemun-gu, Seoul, Republic of Korea

**Keywords:** Epidemiology, Risk factors, Viral infection

## Abstract

The evidence for the impact of benzodiazepine (BZD) use on infection or clinical outcomes of severe acute respiratory syndrome coronavirus 2 (SARS-CoV-2) is limited. We evaluated the association of BZD use with SARS-CoV-2 infection and the clinical outcomes of coronavirus disease 2019 (COVID-19) using a nationwide COVID-19 database from South Korea. This nationwide cohort study was performed using the COVID-19 database from the Health Insurance Review and Assessment Service of Korea, and SARS-CoV-2 positivity was investigated according to BZD use. SARS-CoV-2-positive adult patients were assessed in three groups, those who needed hospitalization, those with severe symptoms requiring intensive care, and those who died. A multivariate logistic regression model was used for all the analyses. After adjusting for potential confounding factors, there was no association between BZD use and SARS-CoV-2 positivity. SARS-CoV-2-positive patients with BZD use showed an increased risk of need for hospitalization from COVID-19 compared to those without BZD use (odds ratio [OR]: 1.33, 95% confidence interval [CI] 1.07–1.65). In addition, there was a higher risk for long-term users (OR: 2.64, 95% CI 1.08–6.47). Chronic BZD use contributed to a higher risk of the need for hospitalization among COVID-19 patients, whereas BZD use did not increase the risk of SARS-CoV-2 test positivity, severe outcomes, or mortality.

## Introduction

The coronavirus disease (COVID-19) pandemic is causing a global crisis. COVID-19 is caused by severe acute respiratory syndrome coronavirus 2 (SARS-CoV-2), with consequences ranging from asymptomatic disease to death^1^. However, 14% of SARS-CoV-2-positive patients show severe disease, and 5% show critical health conditions^2^; the risk of mortality (0.1%) associated with COVID-19 is much higher than that associated with seasonal influenza^3^. Various risk factors such as age ≥ 65 years, chronic obstructive pulmonary disease (COPD), asthma, hypertension, cardiovascular disease, chronic kidney disease, diabetes, obesity, malignancy, immunosuppressant use and transplantation, and chronic human immunodeficiency virus (HIV) infection have been identified^3^. In addition, patients with a mental illness were also shown to have a higher risk for severe COVID-19 outcomes^4,5^.

Benzodiazepines (BZD) and BZD receptor agonists have been known to increase the risk of pneumonia and death due to pneumonia^6,7^. There is also concern about the risk of respiratory depression by BZD use in people with pre-existing respiratory problems, although reports have been conflicting; an increased risk of respiratory exacerbations was reported for patients with COPD^8^, while associations of BZD use with hospital admission or impaired blood gases were not significant in COPD patients^9^. A few recent studies on the characteristics of severe COVID-19 cases^10–12^ have focused on the use of BZD. However, the results have been inconsistent.

BZD were developed and have been widely prescribed for treating anxiety and insomnia, however, recent studies on the prescription trends have shown that BZD use has been increased for so many different indications^13,14^. Given the high prevalence (2.6–23.7%) of BZD use^14–19^, investigating the impact of BZD use on infection or clinical outcomes of SARS-CoV-2 is beneficial for public health. Here, we assessed the association of BZD use with SARS-CoV-2 positivity and clinical outcomes in three groups of patients, those in need of hospital admission, those with severe symptoms requiring intensive care, and those who died, using a nationwide cohort data from South Korea.

## Methods

### Study design and population

In this study, National Health Information Database (NHID)-COVID data provided by the National Health Insurance Service (NHIS) were used; data pertaining to the period from 2015 to 2020 were obtained. The dataset included data from patients with COVID-19 who tested positive from January 1 to May 30, 2020. Control groups included general controls and subjects who showed negative SARS-CoV-2 test results.

**Figure 1:**
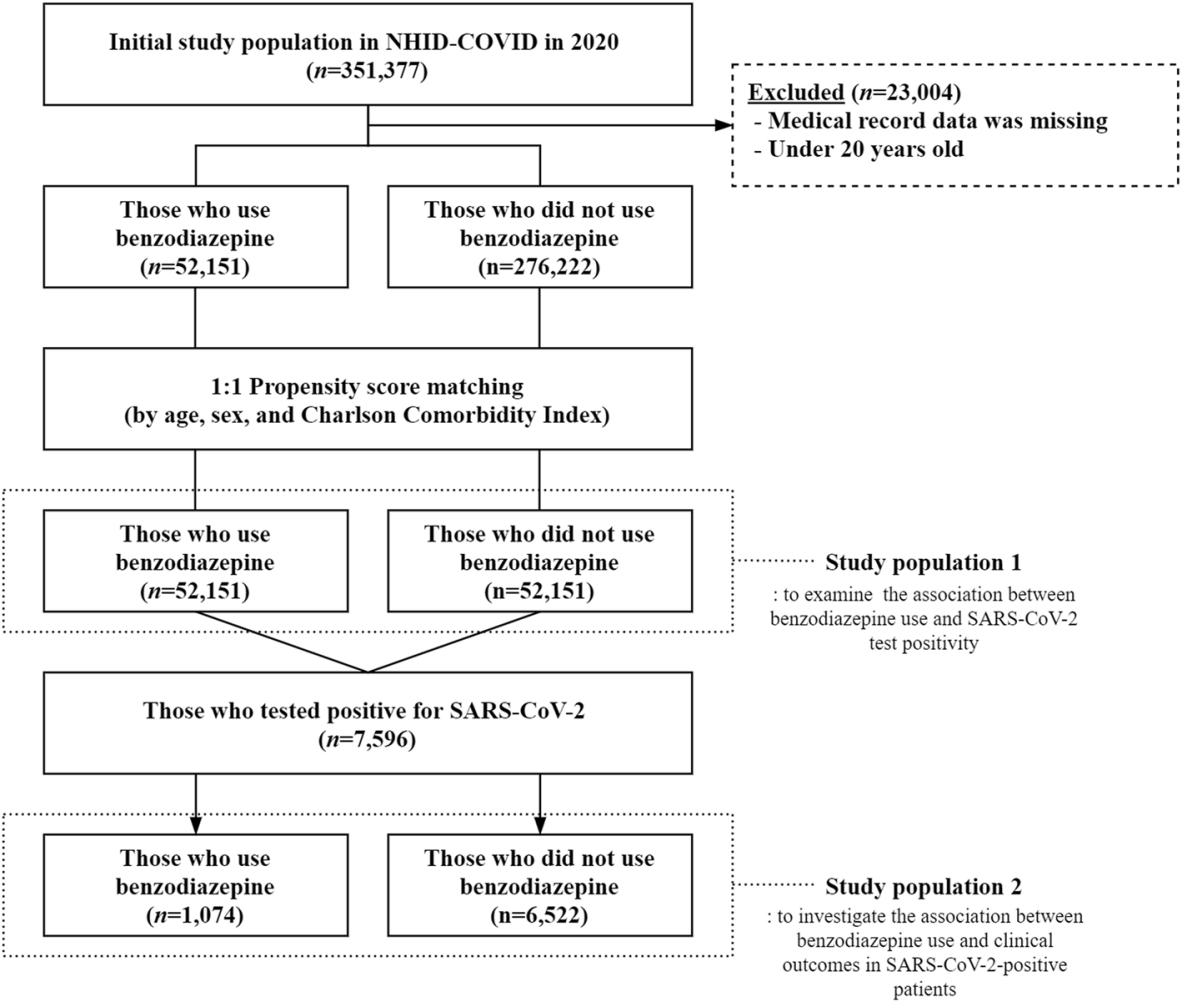
Flowchart showing the selection of the study population. *NHID-COVID* National Health Information Database-Coronavirus disease, *SARS-CoV-2* severe acute respiratory syndrome coronavirus 2.

There are two different study population in this study. Study population 1 is to examine the association between benzodiazepine use and SARS-CoV-2 test positivity, and study population 2 is to investigate the association between benzodiazepine use and clinical outcomes in SARS-CoV-2-positive patients. The initial population for the year 2020 included a total of 351,377 subjects. These were categorized into 8070 patients with SARS-CoV-2, and 343,307 controls. After excluding those with missing values, 328,373 subjects remained. They were divided into two different groups according to whether BZD was used or not: 52,151 subjects used BZD and 276,222 subjects did not use BZD. Using the 1:1 propensity score matching (PSM) method, 52,151 participants each from the case group and the control group were selected as the final study subjects (out of a total of 104,302 subjects). In addition, a total of 7596 patients with COVID-19 were divided into two groups according to whether BZD used or not: 1074 subjects used BZD and 6522 subjects did not use BZD (Fig. 1).

### Measures

#### Outcome variables

In this study, we set SARS-Cov-2 test positivity and clinical outcomes as the outcome variables. The clinical outcomes for SARS-CoV-2-positive patients consisted of three variables: hospital admission, severe symptoms requiring intensive care or invasive ventilation, and mortality. There have been cases of patients who died before receiving hospital care. They did not receive any treatment according to the data records, however, it was assumed that they needed treatment after SARS-CoV-2 infection but died before receiving it. Therefore, they were included in the group that required hospital admission.

#### Variables of interest

The study subjects were divided into two groups according to BZD use. Use of BZD was defined based on claim history in 2019, one year before the outbreak of COVID-19 in 2020.

BZD categories were based on the following main ingredient codes: clobazam (135702ATB), clonazepam (136401ATB), chlordiazepoxide (131201ATB, 131202ATB, 255800ATB), diazepam (142930BIJ), flunitrazepam (160601ATB), flurazepam (161801ATB), ethyl loflazepate (156201ATB, 156202ATB), alprazolam (105501ATB, 105502ATB, 105504ATB, 105505ATB, 105507ATB), bromazepam (118501ATB), clotiazepam (137302ATB), etizolam (156501ATB, 156502ATB, 156503ATB), lorazepam (185501ATB, 185504ATB), tofisopam (241201ATB), and triazolam (243501ATB, 243502ATB). In addition, chronic BZD use was categorized based on a 90-day usage period and a 180-day usage period within a year^14,20^.

#### Covariates

In this study, we adjusted for variables that could directly or indirectly affect outcomes, including basic independent variables such as sex, age, and residential area. The residential areas were grouped based on the major regions and the metropolitan areas where the incidence of COVID-19 was high in Korea at that time. Gyeonggi-do province is the metropolitan area that surrounds Seoul, and Gyeongbuk province and Daegu city are the regions that caused a social issue due to a cluster infection related to the COVID-19 outbreak. Accordingly, Seoul, Gyeonggi-do, Gyeongbuk, and Daegu were classified into each group, and the rest of the regions were combined into a single group. Type of insurance coverage was classified into three different groups: workplace, local, and medical benefits. Participants’ clinical baseline characteristics were also considered as covariates. The Charlson Comorbidity Index (CCI) was used to confirm the patients’ comorbidity status in 2019, which was the year prior to the COVID-19 outbreak. Based on CCI data, the severity of comorbidity was categorized as 0, 1, or 2+. Additionally, we reviewed the disease records for the period from 2015 to 2018 for diabetes, cardiovascular disease, cerebrovascular disease, COPD, asthma, hypertension, and chronic kidney disease, which could be associated with worse clinical outcomes and BZD use.

### Statistical analysis

We investigated the results after 1:1 PSM; in this method, the case and control participants who have a similar propensity score values are matched^21^. We matched case and control groups by including age, sex, and CCI variables as parameters in the propensity score model. The association between BZD use and SARS-CoV-2 positivity was examined in a total of five models. Model 1 was a crude model for the association between BZD use and SARS-CoV-2 positivity; model 2 was a minimally adjusted model adjusted for age and sex; model 3 was the fully adjusted model, which was adjusted for age, sex, residential area, type of insurance coverage, CCI, and disease history (diabetes, cardiovascular disease, cerebrovascular disease, COPD, asthma, hypertension, and chronic kidney disease); model 4 was the fully adjusted model for the association between the chronic use of BZD for 90 days and SARS-CoV-2 positivity; and model 5 was the fully adjusted model for the association between the chronic use of BZD for 180 days and SARS-CoV-2 positivity. Among patients who tested positive for SARS-CoV-2, Pearson's chi-square tests were used to compare the sociodemographic and clinical characteristics with respect to BZD use and chronic BZD use. To examine the association of BZD use and the chronic BZD use with the risk of SARS-CoV-2 positivity and clinical outcomes, we used multivariable logistic regression models after adjusting for sex, age, residential area, CCI, and disease history.

All the statistical analyses were performed using SAS statistical software version 9.4 (Statistical Analysis System Institute, Cary, NC, USA).

### Ethics approval and consent to participate

The data were anonymized before they were obtained; thus, informed consent was not required. The Yonsei University Institutional Review Board approved this study (Approval number: 4-2020-1240). All methods were performed in accordance with the relevant guidelines and regulations.

## Results

The characteristics of the study participants are shown in Table 1. A total of 328,373 individuals were divided into two groups as follows: those who did not use BZD (n = 276,222) and those who used BZD (n = 52,151). The two groups were matched by propensity scoring, and 52,151 propensity-matched pairs were defined (eTable S1). Of the total subjects, 145,758 (44.4%) were men and 182,615 (55.6%) were women. A majority of the participants (84.1%) was aged 20–39 years; 31.1% were in the 40–59 age group. Comorbidities were recorded in 193,945 (59.1%) individuals, while no comorbidities were observed in 134,428 (40.9%) individuals.

**Table 1:** General characteristics of the study population.

Variables	Overall participants	
	N	%
Total	328,373	100.0
**Benzodiazepine use**		
No	276,222	84.1
Yes	52,151	15.9
**Age**		
20–39	121,707	37.1
40–59	102,102	31.1
60–79	77,042	23.5
80 +	27,522	8.4
**Sex**		
Male	145,758	44.4
Female	182,615	55.6
**Residential area**		
Seoul	54,069	16.5
Gyeonggi-do	101,327	30.9
Daegu	57,779	17.6
Gyeongbuk	27,271	8.3
Others	87,927	26.8
**Type of insurance coverage**		
NHI (community)	79,799	24.3
NHI (workplace)	232,470	70.8
Medical aid	16,104	4.9
**CCI**		
0	134,428	40.9
1	154,646	47.1
2 +	39,299	12.0
**History of diabetes**		
No	266,827	81.3
Yes	61,546	18.7
**History of cardiovascular disease**		
No	300,582	91.5
Yes	27,791	8.5
**History of cerebrovascular disease**		
No	301,142	91.7
Yes	27,231	8.3
**History of COPD**		
No	316,520	96.4
Yes	11,853	3.6
**History of asthma**		
No	271,097	82.6
Yes	57,276	17.4
**History of hypertension**		
No	241,649	73.6
Yes	86,724	26.4
**History of chronic kidney disease**		
No	320,270	97.5
Yes	8,103	2.5

All individual characteristics were surveyed as of 2020, and the last survey was recorded until the end of May 2020.

*NHI* National health insurance, *CCI* Charlson Comorbidity Index, *COPD* chronic obstructive pulmonary disease.

Table 2 shows the association between BZD use and the risk of SARS-CoV-2 positivity; we identified these associations before and after adjusting for potential confounders. After PSM, there was no association between BZD use and the risk of SARS-CoV-2 positivity (model 1: odds ratio [OR]: 1.01, 95% confidence interval [CI] 0.93–1.10, model 2: OR: 1.01, 95% CI 0.93–1.10, model 3: OR: 1.09, 95% CI 1.00–1.20). Moreover, model 4 and 5 showed that there were no associations between chronic BZD use and the risk of SARS-CoV-2 positivity (model 4: OR: 0.94, CI 0.81–1.10, model 5: OR: 0.99, CI 0.83–1.18).

**Table 2:** The results of the analysis of the association between benzodiazepine use and severe acute respiratory syndrome coronavirus 2 (SARS-CoV-2) positivity after 1:1 propensity score matching.

Variables	SARS-CoV-2 test positivity																			
	Model 1				Model 2				Model 3				Model 4				Model 5			
	OR	95% CI			OR	95% CI			OR	95% CI			OR	95% CI			OR	95% CI		
**Benzodiazepine use**																				
No	1.00		–		1.00		–		1.00		–		1.00		–		1.00		–	
Yes	1.01	0.93	–	1.10	1.01	0.93	–	1.10	1.09	1.00	–	1.20	0.94	0.81	–	1.10	0.99	0.83	–	1.18
**Age**																				
20–39	–				1.00		–		1.00		–		1.00		–		1.00		–	
40–59	–				0.91	0.82	–	1.01	0.69	0.62	–	0.77	0.69	0.62	–	0.77	0.69	0.62	–	0.77
60–79	–				0.39	0.35	–	0.44	0.31	0.27	–	0.35	0.31	0.27	–	0.35	0.31	0.27	–	0.35
80 +	–				0.47	0.41	–	0.55	0.47	0.39	–	0.56	0.47	0.39	–	0.56	0.47	0.39	–	0.56
**Sex**																				
Male	–				1.00		–		1.00		–		1.00		–		1.00		–	
Female	–				1.36	1.23	–	1.49	1.24	1.13	–	1.36	1.24	1.13	–	1.36	1.24	1.14	–	1.36
**Residential area**																				
Seoul	–				–				1.00		–		1.00		–		1.00		–	
Gyeonggi–do	–				–				5.37	4.48	–	6.44	5.34	4.45	–	6.40	5.34	4.46	–	6.41
Daegu	–				–				0.84	0.65	–	1.08	0.84	0.65	–	1.08	0.84	0.65	–	1.08
Gyeongbuk	–				–				3.99	3.24	–	4.91	3.98	3.23	–	4.90	3.98	3.24	–	4.90
Others	–				–				0.83	0.66	–	1.04	0.83	0.66	–	1.04	0.83	0.66	–	1.04
**Type of insurance coverage**																				
NHI (community)	–				–				1.00		–		1.00		–		1.00		–	
NHI (workplace)	–				–				0.84	0.76	–	0.93	0.84	0.76	–	0.93	0.84	0.76	–	0.93
Medical aid	–				–				1.55	1.31	–	1.83	1.57	1.33	–	1.86	1.56	1.32	–	1.85
**CCI**																				
0	–				–				1.00		–		1.00		–		1.00		–	
1	–				–				1.08	0.97	–	1.20	1.07	0.96	–	1.20	1.08	0.96	–	1.20
2 +	–				–				1.08	0.93	–	1.26	1.07	0.92	–	1.25	1.07	0.92	–	1.25
**History of diabetes**																				
No	–				–				1.00		–		1.00		–		1.00		–	
Yes	–				–				1.04	0.92	–	1.17	1.04	0.93	–	1.17	1.04	0.92	–	1.17
**History of cardiovascular disease**																				
No	–				–				1.00		–		1.00		–		1.00		–	
Yes	–				–				0.97	0.82	–	1.14	0.98	0.83	–	1.15	0.97	0.83	–	1.15
**History of cerebrovascular disease**																				
No	–				–				1.00		–		1.00		–		1.00		–	
Yes	–				–				0.98	0.85	–	1.14	0.99	0.85	–	1.16	0.99	0.85	–	1.15
**History of COPD**																				
No	–				–				1.00		–		1.00		–		1.00		–	
Yes	–				–				0.87	0.68	–	1.11	0.87	0.68	–	1.11	0.87	0.68	–	1.11
**History of asthma**																				
No	–				–				1.00		–		1.00		–		1.00		–	
Yes	–				–				0.94	0.84	–	1.05	0.95	0.85	–	1.06	0.95	0.85	–	1.06
**History of hypertension**																				
No	–				–				1.00		–		1.00		–		1.00		–	
Yes	–				–				1.03	0.92	–	1.16	1.04	0.92	–	1.17	1.03	0.92	–	1.17
**History of chronic kidney disease**																				
No	–				–				1.00		–		1.00		–		1.00		–	
Yes	–				–				0.72	0.51	–	1.02	0.71	0.50	–	1.01	0.71	0.50	–	1.01

Model 1: crude model; Model 2: minimally adjusted; Model 3: fully adjusted; Model 4: chronic use of BZD for 90 days; Model 5: chronic use of BZD for 180 days.

*NHI* National health insurance, *CCI* Charlson Comorbidity Index, *COPD* chronic obstructive pulmonary disease.

Table 3 shows the distribution of the study population who tested positive for SARS-CoV-2. Of 7596 individuals, 1074 (14.1%) used BZD and 6522 (85.9%) did not use BZD. A total of 6019 individuals who were in need of hospitalization, 939 (15.6%) individuals used BZD. A total of 464 individuals who were in need of intensive care or invasive ventilation, 111 (23.9%) individuals used BZD. A total of 233 individuals who died, 59 (25.3%) individuals used BZD.

**Table 3:** General characteristics of the patients who tested positive for severe acute respiratory syndrome coronavirus 2 (SARS-CoV-2).

Variables	Total		Hospital admission^a^		Severe outcome^b^		Mortality	
	N	Column %	N	Column %	N	Column %	N	Column %
Total (N, Row %)	7596	100.0	6019	79.2	464	6.1	233	3.1
**Benzodiazepine use**								
No	6522	85.9	5080	84.4	353	76.1	174	74.7
Yes	1074	14.1	939	15.6	111	23.9	59	25.3
**Age**								
20–39	2839	37.4	1975	32.8	58	12.5	1	0.4
40–59	2563	33.7	2026	33.7	99	21.3	16	6.9
60–79	1795	23.6	1622	26.9	216	46.6	100	42.9
80 +	399	5.3	396	6.6	91	19.6	116	49.8
**Sex**								
Male	2992	39.4	2464	40.9	250	53.9	131	56.2
Female	4604	60.6	3555	59.1	214	46.1	102	43.8
**Residential area**								
Seoul	501	6.6	493	8.2	20	4.3	3	1.3
Gyeonggi-do	4971	65.4	3521	58.5	234	50.4	150	64.4
Daegu	422	5.6	415	6.9	41	8.8	14	6.0
Gyeongbuk	921	12.1	837	13.9	101	21.8	50	21.5
Others	781	10.3	753	12.5	68	14.7	16	6.9
**Type of insurance coverage**								
NHI (community)	2048	27.0	1606	26.7	115	24.8	66	28.3
NHI (workplace)	4898	64.5	3855	64.0	293	63.1	125	53.6
Medical aid	650	8.6	558	9.3	56	12.1	42	18.0
**CCI**								
0	3559	46.9	2584	42.9	108	23.3	22	9.4
1	3291	43.3	2749	45.7	249	53.7	116	49.8
2 +	746	9.8	686	11.4	107	23.1	95	40.8
**History of diabetes**								
No	6409	84.4	4917	81.7	291	62.7	108	46.4
Yes	1187	15.6	1102	18.3	173	37.3	125	53.6
**History of cardiovascular disease**								
No	7192	94.7	5635	93.6	407	87.7	183	78.5
Yes	404	5.3	384	6.4	57	12.3	50	21.5
**History of cerebrovascular disease**								
No	7117	93.7	5554	92.3	395	85.1	162	69.5
Yes	479	6.3	465	7.7	69	14.9	71	30.5
**History of COPD**								
No	7456	98.2	5887	97.8	435	93.8	200	85.8
Yes	140	1.8	132	2.2	29	6.3	33	14.2
**History of asthma**								
No	6541	86.1	5137	85.3	366	78.9	172	73.8
Yes	1055	13.9	882	14.7	98	21.1	61	26.2
**History of hypertension**								
No	6016	79.2	4545	75.5	249	53.7	67	28.8
Yes	1580	20.8	1474	24.5	215	46.3	166	71.2
**History of chronic kidney disease**								
No	7528	99.1	5953	98.9	447	96.3	213	91.4
Yes	68	0.9	66	1.1	17	3.7	20	8.6

*NHI* National health insurance, *CCI* Charlson Comorbidity Index, *COPD* chronic obstructive pulmonary disease, *ICU* intensive care unit.

^a^Hospital admission comprised admission, admission to the intensive care unit, invasive ventilation, or mortality.

^b^Severe outcome comprised admission to the intensive care unit or invasive ventilation.

**Figure 2:**
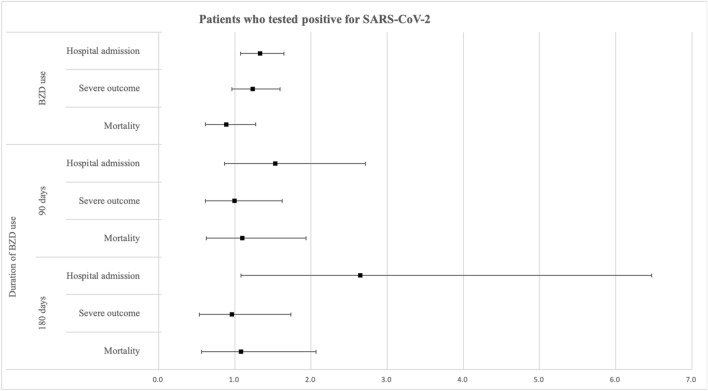
The results of the analysis of the association between benzodiazepine use and clinical outcomes. *BZD* benzodiazepine.

Figure 2 shows the association between BZD use and the clinical outcomes of COVID-19 among patients who tested positive for SARS-CoV-2. After adjusting for potential cofounding variables, the risk of need for hospitalization due to COVID-19 was higher in those with BZD use that in those without BZD use (OR: 1.33, 95% CI 1.07–1.65). In addition, the risk of need for hospitalization was higher in COVID-19 patients who used BZD for more than 180 days than in those who did not use BZD (OR: 2.64, 95% CI 1.08–6.47).

## Discussion

Using a nationwide cohort database from South Korea, in this study, we showed that the chronic use of BZD contributed to an increase in the risk of the need for hospitalization among COVID-19 patients. However, BZD use did not significantly influence the risk of SARS-CoV-2 positivity, severe outcomes, or mortality.

In animal studies, BZD increased mortality due to a variety of bacterial infections^22–25^ and bacterial superinfections related to influenza^22^. In human subjects, controversy persists regarding a causal connection between BZD use and infections^26^. The association between BZD use and the increased need for hospitalization in this study may be in line with previous studies which showed increased susceptibility to superinfections in influenza-infected animals^27^ and humans^7^. The underlying mechanism may be related to the effects of BZD on the immune system; BZD amplifies the effect of the gamma-aminobutyric acid receptor in immune cells, which may lead to an immune-suppressant profile^27^. Regarding the long term use of BZD, chronic consumption of BZD was related to the appearance of modified lymphocyte subsets^28,29^. However, relatively few cases of severe COVID-19 and inaccessible variables in our data, such as the dosage of BZD or the difference of health-related behaviors between short-term and chronic BZD users, may require replication of pharmacoepidemiologic research on the relationship between BZD and COVID-19.

Recent studies on the association between mental illness and COVID-19 outcomes have shown a higher risk for severe COVID-19 outcomes in patients with a mental illness, though the analyses did not include adjustment for BZD use^4,5^. Since BZD is frequently prescribed for anxiety symptoms and sleep disturbances, our findings suggest that BZD use should be considered in further studies on the relationship between mental illness and COVID-19 outcomes. Likewise, adjusting for mental illness in future studies in the association between BZD and COVID-19 outcomes would uncover the risk of BZD use regardless of psychiatric diagnoses. However, it is interesting to note that 87.7% of BZD prescriptions were related to non-psychiatric diagnoses in a nationwide cohort study from South Korea^14^.

Due to limited medical resources, especially with reference to negative pressure beds, policies on the priority for hospitalization among COVID-19 patients have been amended. For example, South Korea has introduced a residential treatment center to isolate asymptomatic patients or patients who do not need hospital care. Therefore, patients with moderate-to-severe symptoms may be hospitalized first^30^. In this regard, the results of this nationwide cohort study may be applied to efficiently set strategies for managing COVID-19 patients, based the finding that patients with chronic BZD use need to be monitored frequently due to a high risk of need for hospitalization; however, the strategies should also consider our finding that BZD use does not imply increased severity of clinical outcomes related to COVID-19.

The use of nationwide longitudinal data strengthens the causal relationship established in our study, and the generalizability of our findings, however, some limitations of this study should be acknowledged. First, although a validation study showed the overall agreement of diagnosis at 82.0%^31^, outcomes were identified by diagnostic and procedural codes, and possible misclassifications cannot be ruled out. Second, data about the indication and BZD dose, as well as the hospitalization period were unavailable, which precluded a full assessment. Especially, since BZD have been prescribed for many indications besides anxiety and insomnia, further studies accessing reasons for BZD uses would help in interpretation of the data. Second, although we adjusted multivariable logistic regression models for the CCI and disease history (diabetes, cardiovascular disease, cerebrovascular disease, COPD, asthma, hypertension, and chronic kidney disease), the result should be interpreted with caution because comorbidities, which may affect prognosis and survival, were recorded in 59.1% of participants in this study. Third, obesity, which is one of the identified risk factors of severe clinical outcomes, was not included in our analyses. In the data we used in this study, body mass index (BMI) can be observed through the results of the patient's health screening questionnaire, but the data on the health screening is not only incomplete but also difficult to actually use it for analysis. For example, the timing and interval of the participants’ health screening varies. Fourth, since the organization in charge of NHIS-COVID data only allowed researchers to extract data for the period that we analyzed in this study, we could not include all the COVID-19 patients up to the present. Thus, we may have missed analyzing new clinical outcomes that may have resulted due to various mutations in SARS-CoV-2. Further studies including the type of mutations in SARS-CoV-2 and the vaccination status of participants could provide a more thorough understanding of the relationship between BZD use and clinical outcomes.

In summary, BZD use was not associated with the risk of SARS-CoV-2 positivity, severe outcomes, or mortality. However, BZD use, especially for more than 180 days, conferred a higher risk of need for hospitalization among COVID-19 patients. Health professionals and public health authorities need to be alert about patients with long-term use of BZD, and these patients need to be closely monitored even if they currently do not need hospital care.

## Supplementary Information

Supplementary Information.

## Data Availability

The data that support the findings of this study are available from the National Health Insurance Service in South Korea but restrictions apply to the availability of these data, which were used under license for the current study, and so are not publicly available. Data are however available from the corresponding authors (SKA and ECP) upon reasonable request and with permission of the National Health Insurance Service in South Korea.

## References

[1] Wiersinga WJ, Rhodes A, Cheng AC, Peacock SJ, Prescott HC (2020). Pathophysiology, transmission, diagnosis, and treatment of coronavirus disease 2019 (COVID-19): A review. JAMA.

[2] Wu Z, McGoogan JM (2020). Characteristics of and important lessons from the coronavirus disease 2019 (COVID-19) outbreak in China: Summary of a report of 72 314 cases from the Chinese Center for Disease Control and Prevention. JAMA.

[3] Jordan, R. E., Adab, P. & Cheng, K. (British Medical Journal Publishing Group, 2020).

[4] Jeon, H. L., Kwon, J. S., Park, S. H. & Shin, J. Y. Association of mental disorders with SARS-CoV-2 infection and severe health outcomes: nationwide cohort study. *Br. J. Psychiatry J. Ment. Sci.* 1–8. 10.1192/bjp.2020.251 (2021). 10.1192/bjp.2020.251 33407954

[5] Lee SW (2020). Association between mental illness and COVID-19 susceptibility and clinical outcomes in South Korea: A nationwide cohort study. The Lancet. Psychiatry.

[6] Obiora E, Hubbard R, Sanders RD, Myles PR (2013). The impact of benzodiazepines on occurrence of pneumonia and mortality from pneumonia: A nested case-control and survival analysis in a population-based cohort. Thorax.

[7] Nakafero G, Sanders RD, Nguyen-Van-Tam JS, Myles PR (2016). The association between benzodiazepines and influenza-like illness-related pneumonia and mortality: A survival analysis using UK Primary Care data. Pharmacoepidemiol. Drug Saf..

[8] Vozoris NT (2013). Benzodiazepine use among older adults with chronic obstructive pulmonary disease. Drugs Aging.

[9] Ekström, M. P., Bornefalk-Hermansson, A., Abernethy, A. P. & Currow, D. C. Safety of benzodiazepines and opioids in very severe respiratory disease: National prospective study. *BMJ* **348** (2014). 10.1136/bmj.g445 PMC3906915 24482539

[10] Genet B (2020). COVID-19 in-hospital mortality and use of renin-angiotensin system blockers in geriatrics patients. J. Am. Med. Dir. Assoc..

[11] Poblador-Plou B (2020). Baseline chronic comorbidity and mortality in laboratory-confirmed COVID-19 cases: Results from the PRECOVID study in Spain. Int. J. Environ. Res. Public Health.

[12] Reilev M (2020). Characteristics and predictors of hospitalization and death in the first 11 122 cases with a positive RT-PCR test for SARS-CoV-2 in Denmark: A nationwide cohort. Int. J. Epidemiol..

[13] Agarwal SD, Landon BE (2019). Patterns in outpatient benzodiazepine prescribing in the United States. JAMA Netw. Open.

[14] Oh S-H (2014). In-depth investigation for prescribing trends of benzodiazepines in South Korea. Int. J. Clin. Pharmacol. Ther..

[15] Mellinger GD, Balter MB, Uhlenhuth EH (1985). Insomnia and its treatment: Prevalence and correlates. Arch. Gen. Psychiatry.

[16] Ohayon MM, Caulet M, Priest RG, Guilleminault C (1998). Psychotropic medication consumption patterns in the UK general population. J. Clin. Epidemiol..

[17] Chong, Y., Fryar, C. D. & Gu, Q. *Prescription sleep aid use among adults: United States, 2005–2010*. (US Department of Health and Human Services, Centers for Disease Control and, 2013).

[18] Olfson M, King M, Schoenbaum M (2015). Benzodiazepine use in the United States. JAMA Psychiat..

[19] Cunningham CM, Hanley GE, Morgan S (2010). Patterns in the use of benzodiazepines in British Columbia: Examining the impact of increasing research and guideline cautions against long-term use. Health Policy.

[20] Bushnell GA, Stürmer T, Gaynes BN, Pate V, Miller M (2017). Simultaneous antidepressant and benzodiazepine new use and subsequent long-term benzodiazepine use in adults with depression, United States, 2001–2014. JAMA Psychiat..

[21] Rosenbaum PR, Rubin DB (1983). The central role of the propensity score in observational studies for causal effects. Biometrika.

[22] Sanders RD (2013). Benzodiazepine augmented γ-amino-butyric acid signaling increases mortality from pneumonia in mice. Crit. Care Med..

[23] Laschi A, Descotes J, Tachon P, Evreux J (1983). Adverse influence of diazepam upon resistance to Klebsiella pneumoniae infection in mice. Toxicol. Lett..

[24] Domingues-Junior M, Pinheiro S, Guerra J, Palermo-Neto J (2000). Effects of treatment with amphetamine and diazepam on Mycobacterium bovis-induced infection in hamsters. Immunopharmacol. Immunotoxicol..

[25] Galdiero F (1995). Effects of benzodiazepines on immunodeficiency and resistance in mice. Life Sci..

[26] Brandt J, Leong C (2017). Benzodiazepines and Z-drugs: An updated review of major adverse outcomes reported on in epidemiologic research. Drugs R&D.

[27] Sanders RD (2015). Immune cell expression of GABAA receptors and the effects of diazepam on influenza infection. J. Neuroimmunol..

[28] Lechin F (1994). Peripheral blood immunological parameters in long-term benzodiazepine users. Clin. Neuropharmacol..

[29] Cosentino M (1996). Assessment of lymphocyte subsets and neutrophil leukocyte function in chronic psychiatric patients on long-term drug therapy. Prog. Neuropsychopharmacol. Biol. Psychiatry.

[30] Ladner, D., Katsumasa, H. & Kyuri, K. The Republic of Korea’s first 70 days of responding to the COVID-19 outbreak. *Global Delivery Initiative Case Study* **13** (2020).

[31] Park, E. *et al.* Assessment of level of agreement in disease codes between health insurance claims data and medical records. *Wonju Health Insur. Rev. Assess. Serv.* (2018).

